# Inhibitory and inductive effects of 4- or 5-methyl-2-mercaptobenzimidazole, thyrotoxic and hepatotoxic rubber antioxidants, on several forms of cytochrome P450 in primary cultured rat and human hepatocytes

**DOI:** 10.1016/j.toxrep.2020.08.003

**Published:** 2020-08-12

**Authors:** Atsuko Miyajima, Yukie Kuroda, Kazue Sakemi-Hoshikawa, Makoto Usami, Katsuyoshi Mitsunaga, Tomohiko Irie, Yasuo Ohno, Momoko Sunouchi

**Affiliations:** a Division of Medical Devices, National Institute of Health Sciences, 3-25-26, Tonomachi, Kawasaki, Kanagawa, 210-9501, Japan; b Division of Pharmacology, National Institute of Health Sciences, 3-25-26, Tonomachi, Kawasaki, Kanagawa, 210-9501, Japan; c Graduate School of Veterinary Medicine, Azabu University, 1-17-1, Fuchinobe, Chuo-ku, Sagamihara, Kanagawa, 252–5201, Japan; d School of Pharmaceutical Sciences, Toho University, 2-2-1 Miyama, Funabashi, Chiba, 274-8510, Japan; e Kihara Memorial Yokohama Foundation for the Advancement of Life Sciences, 1-6 Suehiro-cho, Tsurumi-ku, Yokohama, Kanagawa, 230-0045, Japan

**Keywords:** MBI, 2-mercaptobenzimidazole, 4-MeMBI, 4-methyl-2-mercaptobenzimidazole, 5-MeMBI, 5-methyl-2-mercaptobenzimidazole, 4(5)-MeMBI, 4(or 5)-methyl-2-mercaptobenzimidazole, CYP, cytochrome P450, T6βH, testosterone 6β-hydroxylation, EROD, 7-ethoxyresorufin O-deethylation, 3-MC, 3-methylcholanthrene, DMSO, dimethyl sulfoxide, AhR, aryl hydrocarbon receptor, PXR, pregnane X receptor, Benzimidazole, Cytochrome P450, Drug-metabolizing activity, Hepatocyte, Primary culture

## Abstract

Effects of 4-methyl-2-mercaptobenzimidazole (4-MeMBI) and 5-methyl-2- mercaptobenzimidazole (5-MeMBI) on cytochrome P450 (CYP) activity were examined in primary cultured rat hepatocytes. Hepatocytes from male Wistar rats were cultured in the presence of 4-MeMBI or 5-MeMBI (0−400 μM), and the activity of CYPs 3A2/4 (48 and 96 h) and 1A1/2 (48 h) was determined by measuring the activity of testosterone 6β-hydroxylation and 7-ethoxyresorufin O-deethylation, respectively. As a result, 4-MeMBI and 5-MeMBI (≥12.5 μM) inhibited CYP3A2 activity. On the other hand, 4-MeMBI (≥25 μM) and 5-MeMBI (≥100 μM) induced CYP1A1/2 activity, being consistent with the previous *in vivo* results. In a comparative metabolism study using primary cultured human hepatocytes from two Caucasian donors, 4-MeMBI and 5-MeMBI induced the activity of CYPs 3A4 and 1A1/2 with individual variability. It was concluded from these results that 4-MeMBI, 5-MeMBI and MBI caused inhibition of CYP3A2 activity in primary cultured rat hepatocytes, suggesting their potential for metabolic drug-drug interactions. Primary cultured rat and human hepatocytes were considered to be useful for the evaluation of effects of the benzimidazole compounds on their inducibility and inhibitory activities of cytochrome P450 forms.

## Highlights

• 4-MeMBI, 5-MeMBI and MBI inhibit CYP3A2 activity in cultured rat hepatocytes.
• 4-MeMBI and 5-MeMBI, not MBI, induce CYP1A1/2 activity in cultured rat hepatocytes.
• Effects of the chemicals on CYP3A2 suggest metabolic drug-drug interaction potential.
• 4-MeMBI and 5-MeMBI induce CYPs 3A4 and 1A1/2 activity in cultured human hepatocytes.
• Primary cultured hepatocytes are useful in CYP experiments of benzimidazole compounds.

## 1 Introduction

4-Methyl-2-mercaptobenzimidazole (4-MeMBI) and 5-methyl-2-mercaptobenzimidazole (5-MeMBI) are methyl derivatives of 2-mercaptobenzimidazole (MBI), containing thioureylene structure responsible for thyrotoxicity and hepatotoxicity [1]. 4-MeMBI and 5-MeMBI are supplied in a 1:1 mixture, 4(or5)-methyl-2-mercaptobenzimidazole (4(5)-MeMBI) (hereafter, all of which are collectively referred to as methyl-MBIs), and have broad industrial applications, for example as rubber antioxidants, corrosion inhibitors, and copper-plating brighteners, similar to MBI [2]. Consequently, unintentional exposure to methyl-MBIs and MBI occurs through exposure to rubber materials and environmental wastewater, making their toxicological assessment necessary [3,4]. In this context, it has been shown that both methyl-MBIs and MBI caused thyrotoxicity and hepatotoxicity after repeated oral administration in rats [[5], [6], [7]].

There are, however, some differences in their effects on hepatic drug-metabolizing activity between methyl-MBIs and MBI. For example, methyl-MBIs induce CYPs, including CYPs 1A1/2 and 2B1/2, in rat liver microsomes after repeated oral administration, but MBI did not, while both methyl-MBIs and MBI inhibited flavin-containing monooxygenase activity [8]. This difference is of significance because it was considered that 4-MeMBI and 5-MeMBI were metabolized by CYPs 1A and 2B in rat liver microsomes to their desulfurized forms thereby detoxifying them [7,9]. These findings also explain, at least partly, the toxicological counteraction in terms of liver hypertrophy between 4-MeMBI and 5-MeMBI [8], since CYPs induced by the former can detoxify the latter and *vice versa*.

The above findings suggest that the effects of 4-MeMBI and 5-MeMBI on hepatic drug-metabolizing activity is different from typical thioureylene compounds such as methimazole, which strongly inhibits the activity of several human haptic microsomal drug-metabolizing enzymes including CYP3A4 [10]. This is very important because CYP3A4, the human homologue of rat CYP3A2, is a major CYP in the liver [11] and is therefore involved in metabolic drug-drug interactions [12]. However, the effects of 4-MeMBI and 5-MeMBI on CYP3A2/4 were not fully examined; no protein expression changes in CYP3A2 were observed by semi-quantitative western blot analysis in a rat *in vivo* experiment [8]. In addition, possible inducibility of CYP3A2/4 by 4-MeMBI and 5-MeMBI should be taken into account when their effects are examined, as suggested from the inducibility of CYP3A4 by benzimidazole compounds [13].

These metabolic and toxicologic studies of methyl-MBIs and MBI are important because metabolic drug-drug interaction can be further complicated by other modifier of CYP3A4 activity including easily available supplements such as *Terminalia arjuna* products [14]. In addition, benzimidazole compounds has common toxicological effects, such as oxidative stress and apoptosis [15], which can cause mechanistic drug-drug interaction.

In the present study, we examined the effects of 4-MeMBI, 5-MeMBI and MBI on the activity of CYPs 3A2 and 1A1/2 in primary cultured rat hepatocytes, which enable experiments under several exposure conditions with fewer animals than in *in vivo* experiments. Primary rat hepatocytes were cultured for 48 or 96 h in the presence of 4-MeMBI, 5-MeMBI or MBI and the activity of CYPs 3A2 and 1A1/2 was determined by measuring activity of testosterone 6β-hydroxylation (T6βH) [16] and 7-ethoxyresorufin *O*-deethylation (EROD) [17], respectively. CYP1A1/2 was selected for validation of the primary cultured rat hepatocytes as an *in vitro* model of rat liver because it is strongly induced by 4-MeMBI and 5-MeMBI, but not by MBI, *in vivo* [8]. With a limited number of donors, inducibility of 4-MeMBI, 5-MeMBI and MBI in primary cultured human hepatocytes was also examined for comparative metabolism studies.

## 2 Materials and methods

### 2.1 Chemicals

**Fig. 1:**
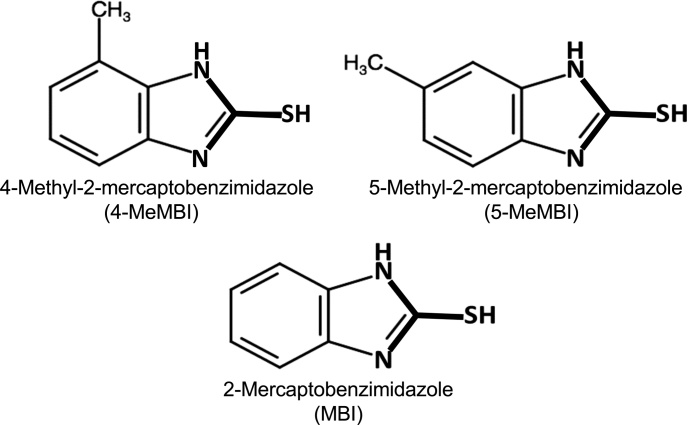
Structures of 4-methyl-2-mercaptobenzimidazole (4-MeMBI), 5-methyl-2-mercaptobenzimidazole (5-MeMBI) and 2-mercaptobenzimidazole (MBI). 4(or 5)-Methyl-MBI (4(5)-MeMBI) is not shown because it is a 1:1 mixture of 4-MeMBI and 5-MeMBI. Thioureylene structure is depicted in bold. Marvin was partly used for drawing the chemical structures [Marvin JS (19.11.0), 2019, ChemAxon (http://www.chemaxon.com)].

The structures of the 4-MeMBI, 5-MeMBI and MBI are shown in Fig. 1. 4(5)-MeMBI (CAS No. 53988-10-6) was supplied from Ohuchi Shinko Chemical Ind., Ltd. (Tokyo, Japan). 5-MeMBI (CAS No. 27231-36-3, PubChem CID: 712373), 3-methylcholanthrene (3-MC, CAS No. 56-49-5) and rifampicin (CAS No. 13292-46-1) were purchased from Sigma-Aldrich Co. LLC. (Merck KGaA, Darmstadt, Germany). 4-MeMBI (CAS No. 27231-33-0, PubChem CID: 3034478) was isolated from 4(5)-MeMBI by repeated fractional recrystallization [18]. MBI (CAS No. 583-39-1, PubChem CID: 707035), dexamethasone (CAS No. 50-02-2) and omeprazole (CAS No. 73590-58-69) were purchased from Wako Pure Chemical Industries (Osaka, Japan). These chemicals were dissolved in dimethyl sulfoxide (DMSO; final concentration 0.1 %) before the addition to the culture medium.

### 2.2 Culture of rat hepatocytes

Rat hepatocytes were prepared by the two-step collagenase perfusion method [19] from male Wistar rats (6-weeks old) purchased from Japan SLC Inc., (Shizuoka, Japan). The hepatocytes were seeded into 24-well culture plates coated with collagen (BioCoat Collagen I 24-well plate, Corning Inc., Corning, NY, USA) at a cell density of 2 × 10^5^ cells/cm^2^ in the Lanford medium (Nissui Pharmaceutical Co., Ltd., Tokyo, Japan), and allowed to attach to the culture plate during pre-incubation for 3 h at 37 °C with 5% CO_2_. After the pre-incubation, the culture medium was replaced with 0.25 mL/well of the Lanford medium containing designated concentration of chemicals. The hepatocytes were cultured in the presence of chemicals for 48 or 96 h, during which the culture medium was replaced every 24 h. All animal experiments were carried out according to the guidelines for animal use of the National Institute of Health Sciences.

### 2.3 Culture of human hepatocytes

Freshly isolated human hepatocytes from two donors, Sau01 (Caucasian male, 17-years old, smoking 1 pack per day for 1 year) and Sau06 (Caucasian female, 17-years old, no smoking history), were purchased (Gentest, Corning) as attached cells to 24-well culture plates (BioCoat Collagen I 24-well plate, Corning) at a cell density of 2 × 10^5^ cells/cm^2^. The hepatocytes were pre-cultured in the Lanford medium, which was replaced every 48 h, at 37 °C with 5% CO_2_ for 1 week prior to the experiments. After the pre-culture, the culture medium was replaced with 0.25 mL/well of the Lanford medium containing designated concentration of chemicals. The hepatocytes were cultured in the presence of chemicals for 48 or 96 h, during which the culture medium was exchanged every 24 h. Effects of MBI on human hepatocytes were not examined because of the scarcity of available cells. The human hepatocytes used in the present study were purchased from the Gentest, and therefore, the study was in accordance with The Declaration of Helsinki developed by the World Medical Association. For the same reason, the code of ethics of the National Institute of Health Sciences is not applicable.

### 2.4 Determination of CYP activity

T6βH activity for CYP3A2/4 was measured as the conversion of testosterone to 6β-hydroxytestosterone by an HPLC method. The cultured hepatocytes were washed two times with PBS(-), and incubated in the presence of testosterone (250 μM, Sigma-Aldrich) for 2 h at 37 °C with 5% CO_2_. The supernatant of the incubation medium was extracted with ethyl acetate containing 1 μM 11α-hydroxyprogesterone (Sigma-Aldrich), an internal standard, dried and re-dissolved in 50 % methanol. The amount of 6β-hydroxytestosterone in the methanol samples was determined with an HPLC system (LC-10A and SPD-M10Avp, Shimadzu Corp., Kyoto, Japan) with an analysis column (Chemcosorb 5-ODS-H, ∅ 6.0 mm × 150 mm (6A), Chemco Scientific Co. Ltd., Osaka, Japan) and a guard column (Chemcosorb 5-OD-UH, ∅ 4.6 mm × 30 mm (W), Chemco Scientific). The analysis conditions were: flow rate, 1 mL/min; injection volume, 100 μL; column temperature, room temperature; mobile phase A, 10 % methanol; mobile phase B, 90 % methanol; gradient, 50 % A for 20 min, 20 % A for 5 min, 5% A for 9 min, and 50 % for 9 min; detection, 240 nm. The retention time was 14.8 min for 6β-hydroxytestosterone, 21.5 min for 11α-hydroxyprogesterone, and 25.9 min for testosterone.

EROD activity for CYP1A1/2 was measured as the conversion of 7-ethoxyresorufin to resorufin by an HPLC method [20]. The cultured hepatocytes were washed two times with PBS(-), and incubated in the presence of 7-ethoxyresorufin (8 μM, Sigma-Aldrich) and dicumarol (10 μM, Sigma-Aldrich) for 30 min at 37 °C with 5% CO_2_. The amount of resorufin in the supernatant (50 μL) of the incubation medium was determined with an HPLC system (LC-10A and SPD-M10Avp, Shimadzu Corp., Kyoto, Japan) with an analysis column (Capcell pak C-18 UG-120, ∅ 4.6 mm × 150 mm, Osaka Soda Co., Ltd., Osaka, Japan) and a guard column (Capcell C-18 UG-120, ∅ 4.0 mm × 10 mm, Osaka Soda).

### 2.5 Statistical analysis

Statistical significance of the differences between the experimental groups was examined by the one-way analysis of variance with the Dunnett's multiple comparison test at probability levels of 5% and 1%.

## 3 Results

### 3.1 Effects on CYP3A2 activity in primary cultured rat hepatocytes

**Fig. 2:**
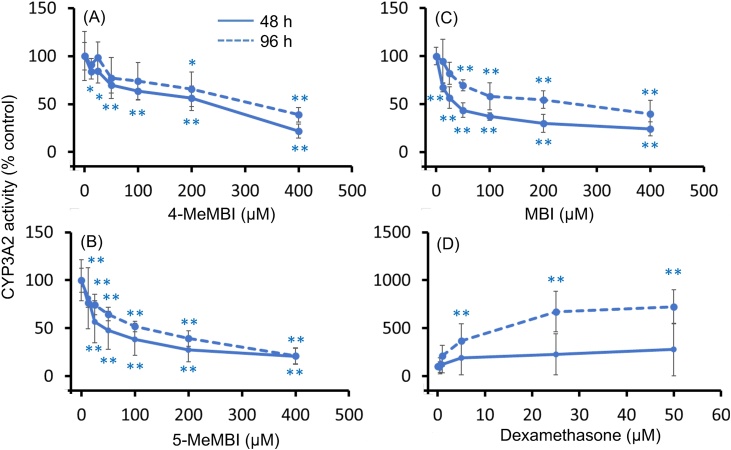
Effects of 4- or 5-methyl-2-mercaptobenzimidazole on CYP3A2 activity in primary cultured rat hepatocytes. Rat hepatocytes were cultured for 48 or 96 h in the presence of chemicals. CYP3A2 activity in the hepatocyte was determined as testosterone 6β-hydroxylation (T6βH) activity by an HPLC method. Data from three experiments were combined after normalization. Mean ± S.E.M. is shown (n = 3-12). The mean activity at 0 μM was 16.04 pmol/10^6^cells/min. Asterisks indicate statistically significant differences compared to the value corresponding to 0 μM (control) (*, p < 0.05; **, p < 0.01).

4-MeMBI, 5-MeMBI and MBI (≥12.5 μM) reduced CYP3A2 activity by more than 50 % in a concentration-dependent manner (Fig. 2A-C). CYP3A2 activity measured at 96 h, however, was generally higher than that at 48 h over the concentration range of 4-MeMBI, 5-MeMBI and MBI used, suggesting an exposure time-dependent weak inducibility of CYP3A2 (Fig. 2A-C). Dexamethasone, a known inducer of CYP3A2, increased CYP3A2 activity at 96 h but not at 48 h, supporting the inducibility of CYP3A2 by 4-MeMBI, 5-MeMBI and MBI in an exposure time-dependent manner (Fig. 2D).

### 3.2 Effects on CYP1A1/2 activity in primary cultured rat hepatocytes

**Fig. 3:**
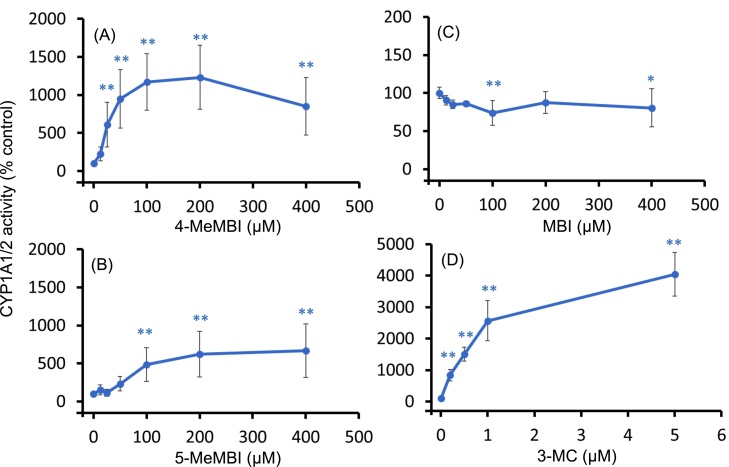
Effects of 4- or 5-methyl-2-mercaptobenzimidazole on CYP1A1/2 activity in primary cultured rat hepatocytes. Rat hepatocytes were cultured for 48 h in the presence of chemicals. CYP1A1/2 activity in the hepatocyte was determined as 7-ethoxyresorufin *O-*deethylation (EROD) activity by an HPLC method. Data from three experiments were combined after normalization. Mean ± s.e.m. is shown (n = 3-18). The mean activity at 0 μM was 1.97 pmol/10^6^cells/min. Asterisks indicate statistically significant differences compared to the value corresponding to 0 μM (control) (*, p < 0.05; **, p < 0.01).

4-MeMBI (≥25 μM) induced CYP1A1/2 activity as much as 12-fold (Fig. 3A). 5-MeMBI (≥100 μM) also increased CYP1A1/2 activity, but only half as much as 4-MeMBI (Fig. 3B). On the contrary, MBI slightly reduced CYP1A1/2 activity over the concentration range used (Fig. 3C). 3-MC, a known inducer of CYP1A, increased CYP1A1/2 activity to a greater extent than 4-MeMBI and 5-MeMBI in a concentration-dependent manner (Fig. 3D).

### 3.3 Effects on CYP3A4 activity in primary cultured human hepatocytes

**Fig. 4:**
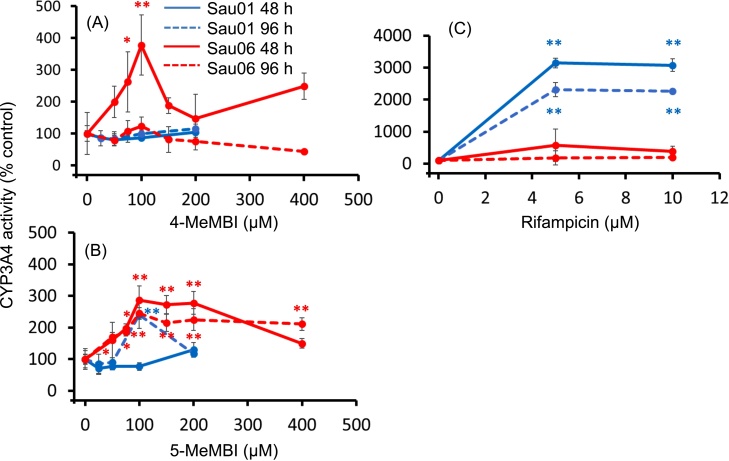
Effects of methyl-2-mercaptobenzimidazole on CYP3A4 activity in primary cultured human hepatocytes. Human hepatocytes from Caucasian donors (Sau01, male, 17 years old, smoking history; Sau06, female, 17 years old, no smoking history) were cultured for 48 or 96 h in the presence of test chemicals. CYP3A4 activity in the hepatocytes was measured as testosterone 6β-hydroxylation (T6βH) activity by an HPLC method. Values of CYP3A4 activity in the hepatocytes from each donor are distinguished by color. The data on Sau01 at 400 μM are not available because of the scarcity of hepatocytes from the donor. Mean ± S.E.M. is shown (n = 3). The mean activity at 0 μM was 6.32 pmol/10^6^ cells/min. Asterisks indicate statistically significant differences compared to the value corresponding to 0 μM (control) (*, p < 0.05; **, p < 0.01).

4-MeMBI increased CYP3A4 activity approximately 4-fold only in Sau06 (75–100 μM, 48 h), suggesting individual variability in its inducibility (Fig. 4A). 5-MeMBI increased CYP3A4 activity approximately 2.5- to 3-fold in Sau01 (100 μM, 96 h) and Sau06 (50–200 μM, 48 h and ≥75 μM, 96 h), suggesting individual variation in inducibility of CYP3A4 and an exposure time-dependency (Fig. 4B). Rifampicin, a known CYP inducer in humans, increased CYP3A4 activity 30-fold only in Sau01 in contrast to 4-MeMBI and 5-MeMBI (Fig. 4C).

### 3.4 Effects on CYP1A1/2 activity in primary cultured human hepatocytes

**Fig. 5:**
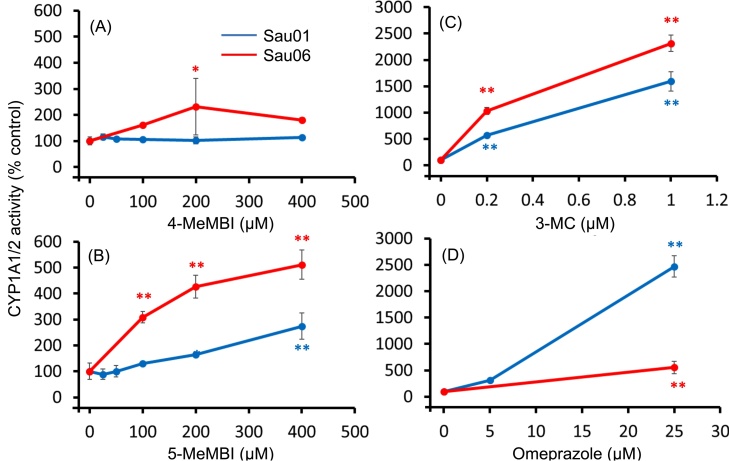
Effects of methyl-2-mercaptobenzimidazole on CYP1A1/2 activity in primary cultured human hepatocytes. Human hepatocytes from two Caucasian donors (Sau01, male, 17 years old, smoking history; Sau06, female, 17 years old, no smoking history) were cultured for 48 h in the presence of test chemicals. CYP1A1/2 activity in the hepatocytes was measured as 7-ethoxyresorufin *O-*deethylation (EROD) activity by the HPLC method. Values of CYP1A1/2 activity in the hepatocytes from each donor are distinguished by color. Mean ± S.E.M. is shown (n = 3). The mean activity at 0 μM was 0.06 pmol/10^6^ cells/min. Asterisks indicate statistically significant differences compared to the value corresponding to 0 μM (control) (*, p < 0.05; **, p < 0.01).

4-MeMBI (200 μM) increased CYP1A1/2 activity in only Sau06, again suggesting that there is individual variability in inducibility in humans (Fig. 5A). 5-MeMBI increased CYP1A1/2 activity in a concentration-dependent manner in both Sau06 (≥100 μM) and Sau01 (400 μM) (Fig. 5B). 3-MC increased CYP1A1/2 activity to a greater extent at much lower concentrations (<1/100) than 4-MeMBI and 5-MeMBI (Fig. 5C). Omeprazole, another known CYP inducer in humans, increased CYP1A1/2 activity to a greater extent in Sau01 than in Sau06, indicating an individual responsiveness opposite to that observed with 4-MeMBI and 5-MeMBI (Fig. 5D).

## 4 Discussion

The present results indicate that 4-MeMBI, 5-MeMBI and MBI inhibit CYP3A2 activity, which can cause metabolic drug-drug interactions. It is considered that the inhibitory effects on CYP3A2 is due to the thioureylene structure, because 4-MeMBI, 5-MeMBI and MBI share this structure, and methimazole, a typical thioureylene compound, inhibits the activity of CYP3A4, the human homolog of rat CYP3A2, in human microsomes [10].

On the other hand, there may be time-dependent weak inductive effects due to the MBI structure as a common profile among 4-MeMBI, 5-MeMBI and MBI, since omeprazole and lansoprazole, antiulcer drugs containing the MBI structure, cause the dual opposite effects on CYP3A4 activity, i.e., inhibition in human microsomes and induction in primary cultured human hepatocytes [13,21].

The above results also indicate the usability of primary cultured rat hepatocytes to examine the effects of 4-MeMBI, 5-MeMBI and MBI on CYP3A2 activity, which might not be observed in *in vivo* studies. This is because these effects were concentration- and time-dependent, and therefore must have required large number of experimental animals if examined in *in vivo* studies. It is suggested that CYP3A2 protein reduced only by MBI in the *in vivo* experiment is due to other factors such as their metabolic profiles; e.g., detoxifying desulfurization was reduced for MBI, but was enhanced for 4-MeMBI and 5-MeMBI, by repeated administration in rats [7,8].

As for CYP1A1/2, 4-MeMBI and 5-MeMBI, but not MBI, induced CYP1A1/2 in the present study; the former was more potent than the latter. These results are consistent with the *in vivo* results where repeated oral administration of either 4-MeMBI or 5-MeMBI induced CYP1A1/2 as determined by EROD and western blot analysis in rat liver microsomes [8]. This consistency validates primary cultured rat hepatocytes as an experimental model for the investigation of 4-MeMBI, 5-MeMBI and MBI.

It is considered that, if any, the different effects on CYPs among 4-MeMBI, 5-MeMBI and MBI both in rat and human hepatocytes were attributable to the presence and position of the methyl group, their sole structural difference. However, there seems no clear relationship between the methylated benzene ring of these chemicals and their inhibitory effects on CYP3A2/4 activity. On the other hand, the different effects of these chemicals confirmed the importance of the substituted benzene ring in the potent inducibility of CYP1A1/2 by benzimidazole compounds in cultured hepatic cells. In cultured rat hepatoma cells, 5-methoxy-MBI induced CYP1A1 protein more potently than MBI [22]. Omeprazole, which has a methoxy group on the benzene ring, induced CYP1A1 more potently than lansoprazole, which has no substituted benzene ring, in cultured hepatic cells [13,22]. Since the inducibility of CYP1A1 by omeprazole depends on the activation of aryl hydrocarbon receptor (AhR), it is supposed that methylated benzene ring strengthens the potency of methyl-MBIs to activate AhR in an order of 4-methyl > 5-methyl in rats, but 5-methyl > 4-methyl in humans.

When rat and human hepatocytes are compared, there seem to be differences in the effects of 4-MeMBI, 5-MeMBI and MBI on CYP activity. This is because inhibitory effects on CYP3A2 of 4-MeMBI and 5-MeMBI observed in rat hepatocytes, were not observed in human hepatocytes. On the other hand, the inducibility of CYP1A1/2 was more potent by 4-MeMBI than by 5-MeMBI in rat hepatocytes, but 5-MeMBI was more potent in human hepatocytes. These differences may be related to known species differences in the inducibility of CYPs [23].

The present results provide some insights into induction mechanisms of CYPs 3A4 and 1A1/2 in human hepatocytes although inconclusive because of the small number of donors. For example, the higher induction of CYP3A4 by 4-MeMBI and 5-MeMBI in the non-responder to rifampicin (Sau06) suggests that the inducibility is pregnane X receptor (PXR)-independent because rifampicin is a PXR-dependent inducer [24]. Similarly, the higher induction of CYP1A1/2 by 4-MeMBI and 5-MeMBI in the low responder to omeprazole (Sau06) suggests that the inducibility is different from that by omeprazole, an inducer known to involve AhR [25].

In terms of comparative metabolism, the induction of CYP1A1/2 activity by 4-MeMBI and 5-MeMBI in rat versus human hepatocytes suggests enhanced detoxification of these chemicals in humans. This is because repeated administration of these chemicals to rats increases hepatic CYP1A1/2 activity and urinary excretion of less toxic desulfurated metabolites *in vivo* [7,8], and because rat hepatic CYP1A metabolizes these chemicals *in vitro* [9]. It was thus considered that 4-MeMBI and 5-MeMBI could be detoxified by CYP1A1/2 induced in the human liver.

It should be noted, however, that the above findings based on human hepatocytes in the present experiment are inconclusive. This is because the number of human donors is as small as two and because possible loss of responsiveness of CYP3A4 to rifampicin as observed in one donor, although the non-responsiveness could be due to great individual variability [26].

In conclusion, 4-MeMBI, 5-MeMBI and MBI cause inhibition of CYP3A2 in primary cultured rat hepatocytes, suggesting their potential for metabolic drug-drug interactions. Primary cultured rat and human hepatocytes are considered to be useful for the evaluation of effects of the benzimidazole compounds on their inducibility and inhibitory activities of cytochrome P450 forms.

## Funding

This work was partly supported by a Health and Labor Science Research Grant from the Ministry of Health, Labor and Welfare in Japan.

## Declaration of Competing Interest

The authors report no declarations of interest.
